# TRPM8 Channels: Advances in Structural Studies and Pharmacological Modulation

**DOI:** 10.3390/ijms22168502

**Published:** 2021-08-07

**Authors:** Carolina Izquierdo, Mercedes Martín-Martínez, Isabel Gómez-Monterrey, Rosario González-Muñiz

**Affiliations:** 1 Departamento de Biomiméticos, Instituto de Química Médica, Juan de la Cierva 3, 28006 Madrid, Spain; carolina.izquierdog@iqm.csic.es (C.I.); iqmm317@iqm.csic.es (M.M.-M.); 2 Programa de Doctorado en Química Orgánica, Universidad Autónoma de Madrid, 28049 Madrid, Spain; 3 Dipartimento di Farmacia, Università degli Studi di Napoli “Federico II”, Via D. Montesano 49, 80131 Naples, Italy

**Keywords:** TRPM8 channels, expression, structure, agonists, antagonists, clinical trials, pain, cancer

## Abstract

The transient receptor potential melastatin subtype 8 (TRPM8) is a cold sensor in humans, activated by low temperatures (>10, <28 °C), but also a polymodal ion channel, stimulated by voltage, pressure, cooling compounds (menthol, icilin), and hyperosmolarity. An increased number of experimental results indicate the implication of TRPM8 channels in cold thermal transduction and pain detection, transmission, and maintenance in different tissues and organs. These channels also have a repercussion on different kinds of life-threatening tumors and other pathologies, which include urinary and respiratory tract dysfunctions, dry eye disease, and obesity. This compendium firstly covers newly described papers on the expression of TRPM8 channels and their correlation with pathological states. An overview on the structural knowledge, after cryo-electron microscopy success in solving different TRPM8 structures, as well as some insights obtained from mutagenesis studies, will follow. Most recently described families of TRPM8 modulators are also covered, along with a section of molecules that have reached clinical trials. To finalize, authors provide an outline of the potential prospects in the TRPM8 field.

## 1. Introduction

In recent years, a part of the scientific community has invested considerable efforts and attention in the structural and physio(patho)logical characterization of the transient receptor potential protein melastatin family (TRPM), a member of the TRP superfamily [1,2,3]. The TRPM family consists of eight (TRPM1–TRPM8) different channels expressed in a wide range of organs and cell structures of the peripheric, central, and immune systems [4,5,6], where they play important roles in different processes, including temperature, taste, and oxidative stress sensing [4,5,6,7,8,9], ion homeostasis [10], cell death [11], myogenic response [12], and the regulation of vascular tone [13]. 

TRPM8, one of the more studied TRPM channels, is a polymodal, Ca^2+^-permeant, and non-selective cation channel, identified as the physiological sensor of environmental cold [4,14,15]. Initially isolated from prostate cancer cells [16], TRPM8 channels are mainly expressed in a subpopulation of sensitive primary afferent neurons [17], which innervate highly cold-sensitive tissues, including skin, the oral cavity epithelium, teeth, nasal mucosa, tongue, and cornea [18,19,20,21,22,23,24,25]. The channel is also expressed in the visceral tissues innervated by the pelvic or vagal nerves as the bladder, male urinary, and genital tracts [26,27,28], as well as in sperm [29], the colon [30], and pulmonary tissues [31,32]. TRPM8 has also been detected in immune system cells as macrophages [33,34] and bone marrow mesenchymal stem cells (hBM-MSCs), in different regions of the mouse brain [35], and in mitochondria-associated endoplasmic reticulum (ER) membranes [36].

**Figure 1:**
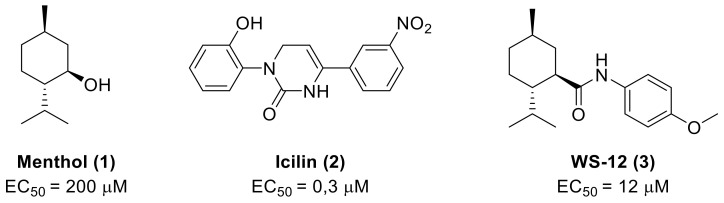
Model TRPM8 agonists (most widely used in pharmacological studies).

TRPM8 channels are activated by a wide range of stimuli, including innocuous to noxious cold temperature (8–26 °C) and cooling agents [4,5,6,33], such as menthol (**1**), icilin (**2**) and the WS-12 menthol analogue (**3**) (Figure 1), voltage [37,38], and changes in extracellular osmolarity [39,40]. The TRPM8 activation/deactivation processes are regulated by genetic [41,42] and structural [43,44,45] modifications, as well as by important endogen factors such as phosphatidylinositol-4,5-bisphosphate (PIP2) [46,47,48], interacting proteins [49,50], G protein-coupled receptor signaling cascades [50,51,52,53], and nuclear receptors [54]. These complex TRPM8 interaction networks are involved in physiological processes, including cold sensation, core body temperature regulation, basal tear secretion, cell differentiation, or insulin homeostasis [4,14,15,25,55,56,57,58]. Consistently, the alteration of its broad expression and function could generate the insurgence or maintenance of pathological processes [59,60]. Functional TRPM8 alterations have been observed in various inflammatory and neuropathic pain states, including dry eye disease (DED) [61,62,63] and migraine [64], oxaliplatin-based cancer peripheral neuropathy [65,66], spinal cord injury [67,68], or after sustained morphine administration [69]. These states are characterized by cold hypersensitivity, which appear as a common symptom, induced by either TRPM8 overexpression or genetic TRPM8 variants [62,66,68,70]. However, the exact role of TRPM8 in the onset of pain in diabetes or multiple sclerosis is still difficult to determine, due to the complexity of signaling pathways and the number of different channels (i.e., K^+^, other TRPs, T calcium channels) that contribute to the neuropathic pain in the corresponding preclinical models [71,72,73,74]. TRPM8 was also linked to other pain types or discomfort states such as irritable bowel syndrome [75,76,77], oropharyngeal dysphagia [78], and chronic cough [79,80], as well as to inflammatory processes of the respiratory tract [81,82], and hypertension [83,84,85]. Last but not least, TRPM8 seems to play an important role in the different steps of cancer progression, including proliferation, apoptosis, and metastasis [14,15,86]. Since the individuation of this channel in prostate cancer cells [16], numerous studies have confirmed the dysregulation of TRPM8 in the plasma of cancer cells in breast [87], bladder [88], esophageal [89], lung [90], human osteosarcoma [91], skin, pancreas, colon, and other gastric cancers [92]. 

This growing body of evidence demonstrates the potential of TRPM8 as a modulator protein in health and specific diseases, although strong contradictions still persist. All the above information, together with recent efforts in the channel structural characterization, constitute a solid support toward the rational design of new efficient/selective modulators. These molecules could also be used as diagnostic probes and/or incorporated alone or in combination in multiplex therapies targeting different cellular and molecular pathways [93,94]. This review article is aimed to recapitulate the latest advances in TRPM8 channels, encompassing, on the one hand, novelties on their distribution and pathological implication, and the big progresses in structural knowledge, driven by cryo-electron microscopy and mutagenesis studies. On the other hand, the review covers newly described agonist and antagonist modulators, their contribution to the pharmacological characterization of the channel, as well as their evolution to clinical trials.

## 2. TRPM8 Distribution and Pathological Implication

A comprehensive review, conceived from a clinical perspective, was recently published by Xiaohui Zhou and coworkers, and compiles publications up to 2019 on TRPM8 distribution and its association with several pathological disorders [95]. As indicated in this review, TRPM8 channels are widely distributed in different tissues and organs, where they are implicated in the regulation of important biological processes. To avoid duplicated information, in this section, we will only focus on 2020–2021 published papers related to the expression of TRPM8 channels and their relationship with different pathological states.

As specified in the previous section, and backed up by an incessant flow of published articles, TRPM8 channel expression is ligated to the progression, migration, and invasion of tumor cells in different cancer malignancies. Over-expression has sometimes been linked to tumor development, while the activation of these channels has protective properties on other tumors, as shown below. Colon cancer liver metastasis has been connected with TRPM8 over-expression, and TRPM8 silencing decreased cell invasion and migration, possibly through the downregulation of p-AKT/AKT, p-GSK3β/GSK3β expression [96]. The evaluation of TRPM8 expression in both prostate normal and pathological tissues, both primary and metastatic secondary tumors, also indicated a higher expression in malignant lesions, especially in the advanced phases of the disease, reinforcing TRPM8 as a prostate biomarker [97]. Similar upregulation of TRPM8 was measured in cutaneous squamous cell carcinoma (SCC), a type of non-melanoma skin cancer (NMSC), while in basal cell carcinoma (BBC) tumors, this channel showed reduced expression [98]. The implication of different types of TRP channels in brain malignant tumors, glioma and glioblastoma, has been reviewed by G. Chinigó, and coworkers [99]. In relation to TRPM8, the interconnection of Ca^2+^ entry by the activation of the TRPM8 channel through the interaction with Ca^2+^-activated K^+^ ion channels (BK channels) was proposed as a possible mechanism. Alternatively, the direct over-expression of TRPM8 channels, as well as the participation of the mitogen-activated protein kinase (MAPK) signaling pathway [100], have also been suggested. The quantification of TRPM8 expression in a clinical study with more than 100 human lung adenocarcinoma samples, correlated channel levels with clinical characteristics and prognosis [101], with high-expression levels of TRPM8 acting as a tumor suppressor, and low levels associated to poor prognostics.

Connected to cancer treatments, it is well known that the administration of oxaliplatin and other chemotherapeutic drugs causes chemotherapy-induced peripheral neuropathy (CIPN), characterized by painful cold allodynia and mechanical hyperalgesia. In a recent article [102], it was demonstrated that oxaliplatin transiently increased the activity of TRPM8 channels 1 h after treatment, while the activity of other members of the TRP channel family was not significantly modified. The activation of phospholipase C (PLC) pathway and the reduction in phosphatidylinositol 4,5-bisphosphate (PIP2) are involved in the possible mechanism. These results point to the pharmacological TRPM8 channel modulation to treat acute and neuropathic pain induced by chemotherapy. 

The increased expression levels of TRPM8 mRNA have been found in interstitial cystitis/bladder pain syndrome (IC/BPS) [103]. In the bladder tissue of IC/BPS patients, the number of TRPM8 neurons and sensory nerves is significantly increased compared to healthy individuals, as shown through immunofluorescence assays. These results point to the implication of TRPM8 channels in the mechanism of bladder pain, the hypersensitivity to pain in IC/BPS patients, and might suggest future alternative treatments for this threatening disease. In fact, some TRPM8 antagonists have been studied on this pain condition, as commented in the corresponding section. TRPM8 channels were also found in the ureter sensory nerve terminals, where they modulate ureter contraction and play an inhibitory role in sensory neurotransmission, through the CGRP-adenyl cyclase-PKA pathway [104]. Mechanical sciatic nerve (MSN) injury in mice produces increased apoptosis, Ca^2+^ entry, and mitochondrial reactive oxygen species (ROS) generation by the nerve lesion. The regeneration of nerve damage can be assisted by heat treatment (through the activation of TRPM2 channels), but cold application did not provide a significant recovery [105]. However, the treatment with a TRPM8 antagonist had a protective role on cold-induced Ca^2+^ influx and on TRPM8 expression in the sciatic nerve, suggesting that changes in Ca^2+^ homeostasis, through the modulation of TRP channels, could play a crucial pathophysiological role in neurons. TRP channels were also found in samples of patients with chronic low back pain, collected after decompressive/fusion spine surgeries. TRPM8 and TRPV4 channels were upregulated in these tissues in contrast to samples from non-symptomatic areas [106]. In cells carrying the Arg30Glu TRPM8 channel mutant, characteristic of familial trigeminal neuralgia, the increased response to menthol (**1**), was observed through Ca^2+^ imaging and Patch-Clamp experiments, indicating that the TRPM8 gain-of-function contributes to pain in this pathology [107]. The predominant involvement of the TRPM8 or TRPA1 channels in cold perception and interspecies dependency is still a matter of controversy. TRPM8 expression, but not TRPA1, was significantly associated to cold-induced pain levels in a cohort of 28 individuals with a high or medium sensitivity to local skin cooling [108], thus, indicating a different contribution of these channel to cold-related persisting pain.

Sensory neurons expressing TRPV1, TRPA1 and TRPM8 have been found in the mouse and monkey cornea, constituting accessible tissue sources to further explore the molecular mechanisms of pathological eye hyperalgesia states. In this respect, a recent article demonstrated that TRPM8 channels are expressed in different populations of terminal corneal C-fibers, separated from those that express TRPV1 and TRPA1, which are co-localized in axons [109].

The cold and menthol-activated receptor TRPM8 is also expressed in subsets of dental primary afferent (DPA) neurons, where it co-localizes with the TRPV1 and Piezo2 channels [110]. In experiments of Ca^2+^ imaging and c-fos expression, the hyperosmolar application of sucrose caused Ca^2+^ transients and c-fos mRNA expression, and both activities were blocked by the TRPM8 antagonist AMTB. These results evidence that TRPM8 channels act as hyperosmosensor, and mediate intense pain (dentin hypersensitivity) caused by sweet foods.

The involvement of the TRPM8 channels as a molecular mediator of human hygrosensation was also recently described [111], with a cold-dry stimulus inducing wellness perception, as it performed the activation by the TRPM8 agonist menthol (**1**), independently of the concurrence of a cooling stimulus or not. TRPM8 channels have also been found at the surface of immune system T-cells, and their activation with the agonist WS-12 (**3**, Figure 1) resulted in T-cell stimulation, with an increase in CD25 and CD69 levels [112].

Although in the lower levels rather than in the peripheral sensory neurons, TRPM8 channels are also expressed in particular areas of the brain, such as the hypothalamus, septum, thalamic reticular nucleus, and brainstem, with similar results in different rodent species [35]. The implication of TRPM8 channels in thermoregulation was suggested too, since positive TRPM8 fibers were identified along the major limbic tract. Related to this, the responses to vascular dilators (bradykinin and glutamate) were reduced after sustained cranial hypothermia compared to normothermic animals [113]. In the last work, the TRPM8 antagonist AMTB inhibited both the endothelium-dependent vasodilation related to hypothermia, and the icilin TRPM8 activation.

Finally, an outstanding article shows the experimental evidence in vitro and in vivo on the regulation of TRPM8 trafficking by axonal organelles and its crucial role for the cold sensation and transduction in peripheral axons [114]. The authors demonstrated the implication of the ARF-GEF Golgi-specific brefeldin A-resistance factor 1 (GBF1) through the inhibition of GBF1 in axons of the sciatic nerve and corneal cold thermoreceptors in vivo. They also provide information about the association of Small GTPase RAB6 with GBF1 protein; thus, identifying two potential targets for indirectly controlling the TRPM8 function in pathological processes, such as neuropathic pain and dry eye disease.

## 3. Insights into TRPM8 Structure 

**Figure 2:**
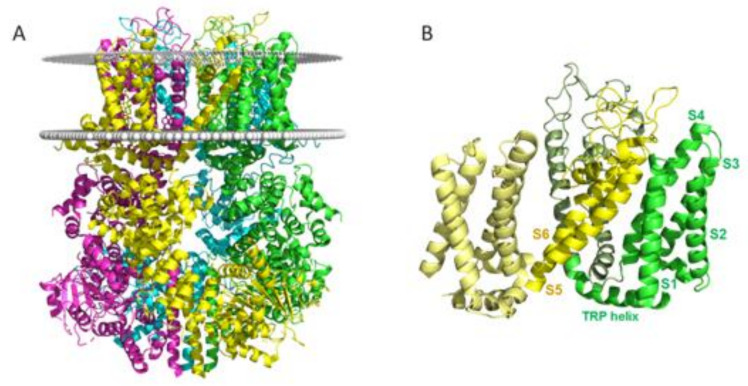
(**A**) Cartoon representation of the full TRPM8 tetramer (based on the structure of pdb code 6O77) [121]. Each subunit is colored differently. (**B**) TM region of two subunits of TRPM8 showing the swapped-domain arrangement. Software for creating images [122,123].

Seok-Yong Lee’s group resolved the first full-length TRPM8 structure from the flycatcher *Ficedula albicollis* (*fa*TRPM8), using cryo-electron microscopy (cryo-EM), with an overall resolution of 4.1 Å (pdb code 6BPQ) [115,116]. The *fa*TRPM8 protein is highly homologous to the human channel, with an 83% sequence identity. The transmembrane (TM) region in this 3D structure has similar architecture to those previously described for TRPV1 and TRPV2 channels [116], with six helical segments. Four of them (S1–S4) form the voltage-sensor-like domain (VSLD), and segments S5 and S6, together with the pore helix, contribute to the pore domain (PD) (Figure 2). The TM region shows a domain-swapped arrangement, in which the VSLD of one protomer interacts with the PD of another. It is worth mentioning that helical features, such us 3_10_ or the π-helix, involved in gating processes in other TRP channels, are absent in this apo-TRPM8 structure. The cytoplasmatic region is composed of the *N*-terminal (NTD) and C-terminal (CTD) domains. Similarly to other TRPM family members, the long NTD comprises four melastatin homology regions (MHR) and the pre-S1 domain. The CTD is composed of a highly conserved region, near of TM S6 (termed the TRP domain, TRPD), three helices and the coiled-coil domain.

The TRP and the VSLD domains form a cavity, termed the VSLD cavity, that had been proposed as the binding pocket for ligands even before the structural determination of cryo-EM TRPM8-ligand complexes. Thus, mutagenesis studies had shown the relevance of certain aromatic residues, as Y745 (human and mouse TRPM8) and Y1005 (human TRPM8, corresponding to Y1004 in *fa*TRPM8). Thus, the Y745A TRPM8 variant is insensitive to menthol, whereas mutant Y745F led to menthol-reduced potency, indicating the importance of both the aromatic ring and the hydroxyl group for channel activation [117,118]. Moreover, studies on [^3^H]menthol affinity assays in membranes expressing the Y745H TRPM8 channel, clearly suggested that the Y745 residue is involved in the direct binding of menthol [119]. Interestingly, this mutant led also to the reduced inhibition of TRPM8 by antagonists as capsazepine, clotrimazole or econazole [120]. 

Aromatic residue Y1005A and Y1005F mutations in mouse TRPM8 (*m*TRPM8), also reduces menthol responses, with a greater decrease for the Y1005A mutant [117]. Mutagenesis studies also showed the importance of charged residues, such as Arg842 (human TRPM8, corresponding to R841 in *fa*TRPM8), since its replacement by Ala, His, and Lys caused a reduced response to menthol [119].

Shortly after the description of the first 3D structure of the apo-*fa*TRPM8, seven new cryo-EM structures were disclosed, including complexes with different ligands and PIP2, and corresponding to TRPM8 channels of two different avian species, *fa*TRPM8 and great tit, *Parus mayor*, TRPM8 (*pm*TRPM8). These new structures help to understand the binding mode of ligands, the requirement of Ca^2+^ for icilin binding, and the location of the PIP2 binding site. As previously suggested by mutagenesis studies, it was shown that the binding pocket for ligands is in a cavity shaped by the VSLD and the TRP domains.

### 3.1. Structures of TRPM8 in Complex with Agonists

Two cryo-EM structures of the *fa*TRPM8 channel bound to the supercooling agonist icilin (**2**) and PPI2, and to menthol analogue WS-12 (**3**) and PIP2, have also been resolved by the group of Seok-Yong Lee [124]. As the avian channels do not respond to icillin, a sensitive mutant, A805G *fa*TRPM8, was used to determine the structure with icilin. The reconstruction of the icilin complex led to two structures, a major class with densities for icilin, PIP2 and Ca^2+^ (3.4 Å, pdb code 6NR3), and another minor with weak or no density for the ligands (4.3 Å, pdb code 6NR4). 

**Figure 3:**
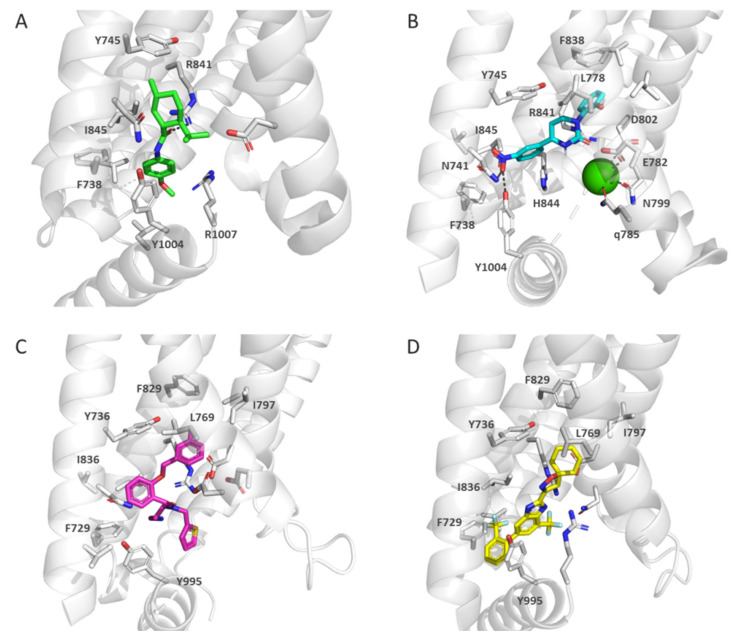
Representation of TRPM8 ligands within the receptor binding site (grey). (**A**) *fa*TRPM8-WS-12 (green) (pdb code 6NR2), (**B**) *fa*TRPM8-Icilin (cyan) (pdb code 6NR3), (**C**) *pm*TRPM8-AMTB (pink) (pdb code 6O6R), (**D**) *pm*TRPM8 TC-I 2014 (yellow) (pdb code 6O72). Residues discussed in the test are labeled. Ca^2+^ is depicted as a green sphere, and hydrogen bonds as dashed grey lines. For clarity, only polar hydrogens are shown. Software for creating images [122].

In the structures generated for the complex of WS-12, PIP2 and *fa*TRPM8 (4 Å, pdb code 6NR2), WS-12 (**3**) binds the VSLD cavity, and interacts with the hydrophobic residues Tyr745, Phe738, Ile845, and Tyr1004 (Figure 3) [124]. Together with Tyr1004, two charged residues, Arg841 and Arg1007, are positioned close to the central WS-12 amide bond. Icilin (**2**) binds in the same cavity in the complex with *fa*TRPM8 (pdb code 6NR3), but both ligands only overlap partially. The icilin molecule becomes surrounded by several hydrophobic residues Tyr745, Phe738, Leu778, Phe838, Ile845, and Tyr1004, with its NO_2_ group forming hydrogen bonds with Asn741 and Tyr1004. In addition, the icilin interacts with two other polar residues, Arg841 and His844, within the cavity (Figure 3). Mutant H844A causes a strong decrease in icilin activation, whereas it does not affect that of the one produced by WS-12. It has been suggested that His844 residue is behind icillin-dependent intracellular pH activation; WS-12 is not pH sensitive. TRPM8 activation by icilin depends on Ca^2+^, and the cryo-EM structure 6NR3 showed the presence of a Ca^2+^ ion coordinated with Glu782, Gln785, Asn799, and Asp802 from *fa*TRPM8 S2 and S3 helices. Before the resolution of this cryo-EM structure, mutagenesis studies had shown the importance of Asn799 and Asp802 in icilin activation, since mutations of these residues had led to icilin-insensitive channels [125]. A similar pattern was found for Ca^2+^ coordination in the structure of *pm*TRPM8 (pdb code 6O77) in which the ion is coordinated by residues, Glu773, Gln776, Asn790 and Asp793 (corresponding to those indicated previously for *fa*TRPM8) [121]. It has been hypothesized that Ca^2+^ might induce a conformational change, allowing icilin binding. In this sense, in the apo form, Asp802 (S3) forms a salt bridge with Arg841 (S4), while the binding of Ca^2+^ causes a slight rotation of the S3 helix, and the break of this salt bridge, placing Asp802 and Asn799 in an appropriate position to coordinate Ca^2+^, and the enlarging of the VSLD cavity [124]. This structure has also provided evidence of the role of a close residue, Gly805 in mammalian TRPM8, which confers sensitivity to icilin, whereas an Ala residue is present in the icilin-insensitive avian channels. This Gly probably provides the necessary flexibility for the rotation of the S3 helix. 

It is also interesting to note that although the structures with icilin/PIP2 and WS-12/PIP2 showed rearrangements comparable to the ligand-free structure, as the rotation of the VSLD and the bent S6, they seemingly represent no conducting states. However, there are also differences in the conformational rearrangements between the complexes with these two agonists. It was speculated that the 6NR3 structure with bound icilin corresponds to a sensitized state of the channel, as PIP2 is fully engaged. In this conformation, there are more interactions among S5, the TRP domain, and a 3_10_-helical segment at S4. Nevertheless, the possibility of a desensitized state due to the high Ca^2+^ concentration used in sample preparation cannot be excluded. On the other hand, it was hypothesized that the WS-12 complex could be a presensitized state, with no significant changes in S5 and a complete α-helix at S4.

These cryo-EM structures also shed light regarding the role of PIP2 in channel activation. In the icilin complex structure (pdb code 6NR3), a strong density corresponding to PIP2 was observed at the interface of the TM domain, the *C*-terminal domain and the fourth MHRs from the adjacent protomer. The PIP2 triphosphate head group interacts with Lys605 (MHR4), Arg688 (pre-S1 domain), Arg650 (S4–S5 linker) and Arg997 (TRP domain). This binding pocket for PIP2 differs from that observed in other TRP channels, as TRPV1 or TRPV5, and it was even suggested that this pocket might not be shared by other TRPM channels, as TRPM4 or TRPM2. On the other hand, the interfacial region in apo-*fa*TRPM8 (6BPQ), and *fa*TRPM8 bound to WS-12 (6NR3) or the low-occupancy icilin (6NR4) are wider compared with that of the high-occupancy icilin (6NR3). In the TRPM8 complex with WS-12, a PIP2 density was observed, but its fitting was less optimal due to the wider cavity. Several structural rearrangements could explain the different dimensions of the PIP2 interfacial cavity. For example, in the icilin-PIP2 bound structure (6NR3), there is a 3_10_-helical segment within an S4 helix, while TM S4 is fully α-helical in the WS-12 complex (6NR2), an upward tilt of the TRP domain and movement of the TM S5. It is worth mentioning that this conformational rearrangement might account for the allosteric role of PIP2 in agonist binding. TRPM8 interaction with PIP2 might favor icilin binding or, alternatively, icilin binding to TRPM8 might induce conformational changes that would help in the PIP2 accommodation. 

### 3.2. Structures of TRPM8 in Complex with Antagonists

The group of D. Julius have resolved the structure of the *pm*TRPM8 complex with two antagonists, AMTB (pdb code 6O6R, 3.6 Å) and TC-I 2014 (pdb code 6O72, 3.2 Å), as well as the apo form (pdb code 6O6A, 3.6 Å) and a complex with Ca^2+^ (pdb code 6O77, 3.6 Å) [121]. The overall structure of the channel was similar to those previously described for *fa*TRPM8. However, the resolution of the *pm*TRPM8 Ca^2+^ bound structure (6O77) allowed determining the PD outer pore loop organization. In this 3D structure, this key loop for channel activity is glycosylated, and has a disulfide bond between the Cys919 and Cys930 residues. Analogously to agonists, antagonists AMTP and TC-1 are accommodated within the cavity formed by the four helices of the VSLD and the TRPD. These antagonists are surrounded by hydrophobic and charged residues, but the lack of ionic interactions suggested shape complementarity as the most important factor for ligand recognition. Both antagonists share several hydrophobic residues within the binding cavity, as Phe729, Tyr736, Ile769, Ile797, Phe829, Ile836, and Tyr995 (Figure 3C,D). The overall structure of TRPM8 in the ligand-free channel and those bound to antagonists is similar; thus, this might indicate that inhibitors lock the channel in the apo conformation.

As the *pm*TRPM8 channel provided high-quality density maps, it was possible to resolve all residues in the TM helical segments, and this led to the description of two TRPM8 conformations—a closed channel, in a complex with antagonists, and a desensitized state in the presence of cold and Ca^2+^. In the close conformation, Met968 and Phe969 provide a restriction in the lower gate. Moreover, in the antagonist complexes, a lipid tail is present in the ion conduction pathway, thus, narrowing the ion path. On the contrary, in the desensitized state, Val966 is the residue that narrows the lower gate. Comparing both states, several conformational rearrangements can be observed between the close and the desensitized states, as the formation of the canonical S4–S5 linker, the stabilization of the PD outer pore loop, and the transition from the α- to π-helix in S6. Moreover, there is a large tilting movement of the TRPD helix, varying its position relative to the VSLD. In this sense, the binding of Ca^2+^ disrupts the salt bridge between the TRPD Arg998 residue and the S2 Gln776, allowing the movement of the TRPD helix. These data support the hypothesis that the desensitization of TRPM8 is due to direct Ca^2+^ binding. Although none of the structures are in the open state, it has been suggested that it can be similar to the desensitized channel, except for the closure of the channel by Val966 [121]. Nevertheless, it cannot be ruled out that it could be different, and not stable enough to be characterized. Interestingly, the mutation of Val966 to Lys changes the selectivity of the channel from cations to anions [126]. 

Regarding the interaction with lipids, in close or desensitized states, they pack around pre-S1 and S1-helix, and the S5 helix of a neighbor subunit, with a higher number in the close state, due to the enlarged cavity [121]. A mixture of lipids was observed, and cholesteryl hemisuccinate (CHS) could be modeled.

The cryo-EM structures have shown that the binding pocket is able to accommodate ligands of different nature, with a variety of substituents. Therefore, the cavity formed by the VSLD and the TRPD helix could adapt to different molecules, which is of interest in the design of novel modulators.

## 4. TRPM8 Agonists

**Figure 4:**
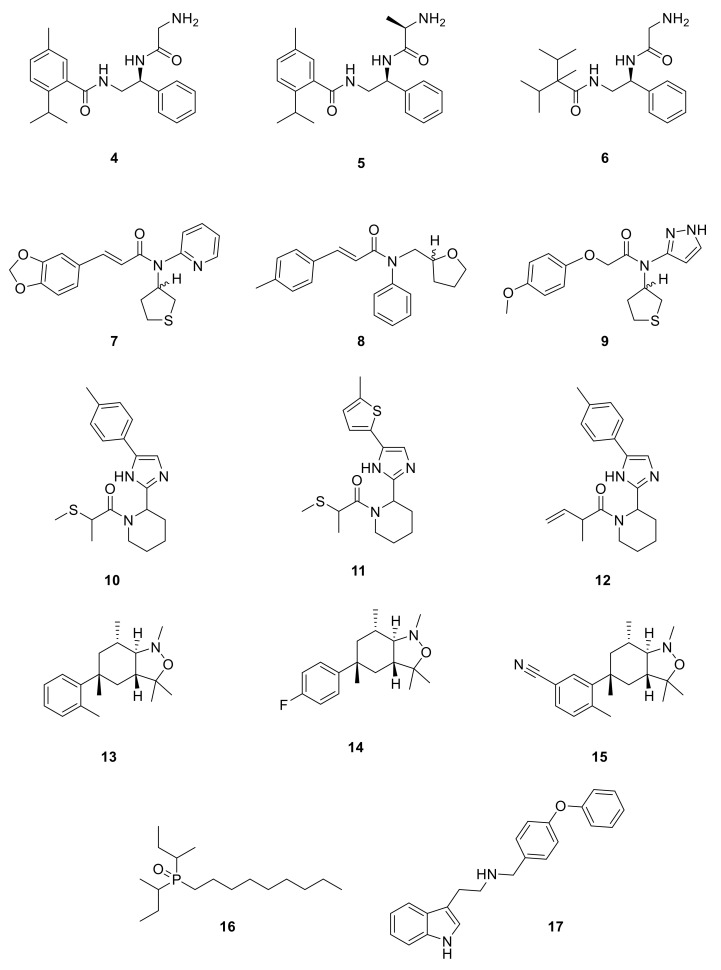
Main TRPM8 agonists: selected compounds from new chemotypes recently described.

Menthol (**1**) and other TRPM8 agonists elicit cooling sensation, making them attractive components for personal care products and as food additives. In recent years, efforts in the search for TRPM8 agonists have been directed primarily towards cooling compounds that overcome the drawbacks produced by natural agonists, as somewhat bitter tasting and irritational. Attention was also focused on the search for the fast-onset and long-lasting cooling effects. The results of these studies are found in patents, claiming the use of these cooling compounds as ingredients in a wide variety of products. Representative compounds, selected from the different families described below, are detailed in Figure 4. Table 1 detailed figures for a representative example of each family.

Procter and Gamble Company directed efforts to find molecules with alternative moieties to the menthol-like cyclohexane fragment [127]. In this patent, they presented a series of derivatives able to activate TRPM8 with EC_50_ values suitable for their use as coolant compounds (EC_50_ ≤ 1 µM). It was also claimed that they can promote thermogenesis, favoring the formation of beige and/or brown adipocytes over white ones and, therefore, of possible application in the treatment of obesity. The biological evaluation showed that phenyl, adamantyl or highly branched alkyl moieties were appropriate replacements of the menthol cyclohexane moiety (Figure 4). Compounds **4** and **5**, containing an aromatic ring instead of the cyclohexane, and a Gly or D-Ala residue, respectively, led to nanomolar agonists (EC_50_ = 1.9 and 3.2 nM, respectively). Slightly higher values were obtained when the menthol-like group was substituted by a diisopropyl methyl group in compound **6** (EC_50_ = 7.8 nM). Similar EC_50_ were obtained for several analogues of compound **5**, as the analogues with an L-Ala residue (6.6 nM) or with L-Ala and a menthol moiety (9 nM). 

BASF SE (US20200190052A1) investigated a series of *N,N*-disubstituted amide derivatives as cooling compounds [128]. Based on a prop-2-enamide or a 3-hidroxipropanoylamide as the central scaffold, they explored a variety of substituents attached at the three possible modification points (**7**–**9**, Figure 4). Three rings have been studied at the left *C-* or *O*-position (1,3-benzodioxol, 4-methylphenyl and 3-methoxyphenyl), whereas a broader range of heterocyclic rings and aliphatic chains has been analyzed as substituents on the amide N atom. Compounds **7**–**9** are within the most potent derivatives in this family, with EC_50_ values of 5–10 µM. In general, these compounds showed a faster onset of the cooling sensation, and a comparable or better profile regarding the overall intensity and long-lasting action compared to the cooling agent FEMA4809 [129]. A study of cold perception at time intervals was also performed, with five derivatives; in particular, compound **7** had a comparable onset to other know cooling compound (WS-3), but a longer cooling effect.

Recently, Givaudan SA disclosed two new families of TRPM8 agonists which differ in the central scaffold. The first family, which described and claimed 208 compounds with EC_50_ below 35 µM, is exemplified by derivatives **10**–**12** (Figure 4) [130]. Almost all of these compounds included a piperidin moiety, although other heterocyclic rings have also been contemplated (azepane, pyrrolidine, and thiazolidine). In a similar way, most of these compounds incorporated an imidazole ring; nevertheless, other heterocyclic derivatives, containing triazole, tetrazole, oxadiazole, isoxazole, and oxazole, have also been prepared. Regarding the substituent on the piperidin nitrogen, the most frequent within potent molecules is the methylthiopropanoyl chain (as in **10** and **11**, Figure 4). Other examples include alkyl (some of them highly branched) or alkene chains, which might or might not contain the sulfur atom. More variety is found in the substituent at position 5 of the imidazole ring, which quite frequently is a phenyl ring with one or more substituents, either equal or different, including methyl (as in **10** and **12**, Figure 4), trifluoromethyl, fluor, chloro, methoxy, amino, nitro, ciano, and propyl. Moreover, the phenyl ring can be replaced by other heteroaromatic rings as thiophene (in **11**), furan, pyridine or pyrimidine, bicyclic heterocyclic moieties, such as difluoro benzo[d][1,3]dioxole, difluoro benzofuran, benzo[b]thiophene or quinoxaline. It is worth to mention that these compounds are chiral, and most of them are described as a mixture of diastereoisomers. Regarding the resolved compound **10**, the corresponding RR or SR diastereoisomers showed agonist activity with EC_50_ values below 0.05 µM, whereas for the SS isomer it was in the range 0.3–0.05 µM. In a test of cooling sensation, compounds **10**, **11** and **12** were considered “strong” coolants regarding maximal cooling (rated from four to eight), and “medium” after an hour (rated from one to four). Similar values were obtained in the analysis of a dentifrice, although in this assay, compound **11** was considered to have a “medium” cooling effect.

Givaudan SA also described a family of 139 molecules containing a heterobicyclic central scaffold, identifying potent derivatives showing EC_50_ values below 35 µM [131,132]. The central skeleton for this library is a benzo[c]isoxazole (as in **13**–**15**, Figure 4). Compounds have been described as racemic mixtures, except for derivative **15** whose enantiomers could be separated, although the assignment of absolute configuration has not been possible. EC_50_ values were in the range 0.5–0.3 μM for the most potent enantiomer and below 5 mM for the other one. Different patterns of substituents on the central bicyclic had widely been explored. Considering the derivatives with EC_50_ values equal or lower to 0.05 µM, the preferred substituents at position one are methyl, ethyl or isopropyl, whereas C3 is disubstituted with two methyl moieties, and position seven also bears a methyl group. A wider range of substituents are found at position five that may be either mono- or disubstituted. At C5, a phenyl (compounds **13** and **14**) or benzonitrile (**15**) are recurrent substituents. In a sensory study, compound **15** was qualified for maximal cooling as “extreme” (score above 8), while **13** and **14** were ranked as “strong”. As for the dentifrice test, **13** and **15** were considered “strong” for maximal cooling, whereas **14** was rated as “medium”. In both assays, after 1 h, these molecules were scored as “medium”. Compounds described in these patents are devoid of significant bitterness or negative organoleptic properties [130,131,132].

Several patents, with E.T. Wei as the inventor, are related to the description of 1-diisopropyl-phosphinoyl-alkanes (such as **16**) as potent TRPM8 agonists and their use as topical agents for sensory distress [133,134], and to treat lower gastrointestinal tract disorders [135]. The best biological profile was found for DIPA derivative with two isopropyl groups and a linear chain of nine carbons (**16,** Cryosim-3, Figure 4), although the corresponding analogues with linear chains from five to eight carbons also provided potent compounds. Significant results were obtained after topical treatment with cryosim-3 of patients with moderate dry eye disease, where no irritation or pain were reported [136]. Very recently, a new patent by the same author claims for the topical application of these phosphinoyl-alkanes cooling compounds in facial skin, as an effective method for alleviating mask discomfort [137]. 

Based on the existing structural information on the modulation of TRPM8 [93,138], Gomez-Monterrey’s group designed a series of tryptamine-based derivatives, including indole ring decoration at the *N*-1, 3, and 5 positions [139]. Data from fluorescence assays, using 0.5, 5, and 50 μM concentrations of synthesized compounds, allowed the identification of derivative **17** (Figure 4) as an agonist of the TRPM8 channels, while related analogues behave as antagonists (see antagonists’ section). In Patch-Clamp electrophysiology assays, the TRPM8 agonist **17** showed a significant potency (EC_50_ = 40 μM) and a similar efficacy compared to menthol (**1**), but it also behaved as a full TRPV1 and TRPA1 antagonist at the highest concentration tested (50 μM). In a chronic constriction injury (CCI) pain model, compound **17** (iv) induced hypersensitivity at a low dose (0.1 mg/0.1 mL), while produced a significant reduction in cold responses at higher doses (0.3 and 1 mg/0.1 mL) [140]. 

Tacrolimus, a macrocyclic immunosuppressant, augments calcium entry in cold-sensitive neurons, through the activation of TRPM8 channels, an activity that can be inhibited by selective TRPM8 antagonists [141]. As expected, this kind of response was lower in TRPM8 *knockout (KO)* mice. Lysophosphatidylcholine (LPC) 18:1 and LPC 16:0, which are over-expressed in oxaliplatin-induced peripheral neuropathy, have been identified as endogenous activators of TRPV1 and TRPM8 channels, suggesting that the modulation of lipid pathways could also serve to alleviate this neuropathy [142].

It is interesting to note in this section, that in 2016, the group of Thomas Voets defined two types of TRPM8 channel agonists: Type I: those that make the closed sate of the channel more stable (i.e., allyl isothiocyanate, AITC); and Type II: those that stabilize the open channel (i.e., menthol, **1**) [143]. To the best of our knowledge, this classification based on distinct state-dependent effects on channel gating has not been considered to date in the description of the new identified agonists, although this could be important for understanding differential actions among agonists.

**Table 1:** Selected examples of newly described families of TRPM8 agonists.

Compd.	Structure	TRPM8 EC_50_^a^ (µM)	Application	Ref.
**4**	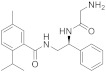	0.0019	Potential treatment for obesity, diabetes, dyslipidemia, irritable bowel syndrome, pain, personal care products or food additive	[127]
**7**	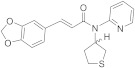	6	Personal care products. In tooth paste, rapid onset and long duration of cooling sensation	[128]
**10**	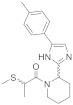	≤0.05	Ranked as “strong” regarding maximal cooling, and “medium” after 1 h	[130]
**15**	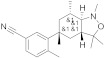	<0.05	Ranked as “extreme” regarding maximal cooling, and “medium” after 1 h	[131,132]
**16**	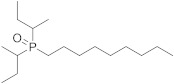	0.9	Sensory and mask discomfort Gastrointestinal tract disorders Pain Dry eye disease	[136,137]
**17**	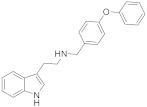	40 ^b^	Pain	[139,140]

^a^ Ca^2+^ fluorimetry assays. ^b^ Patch Clamp experiments in rat TRPM8 (*r*TRPM8).

## 5. TRPM8 Antagonists

**Figure 5:**
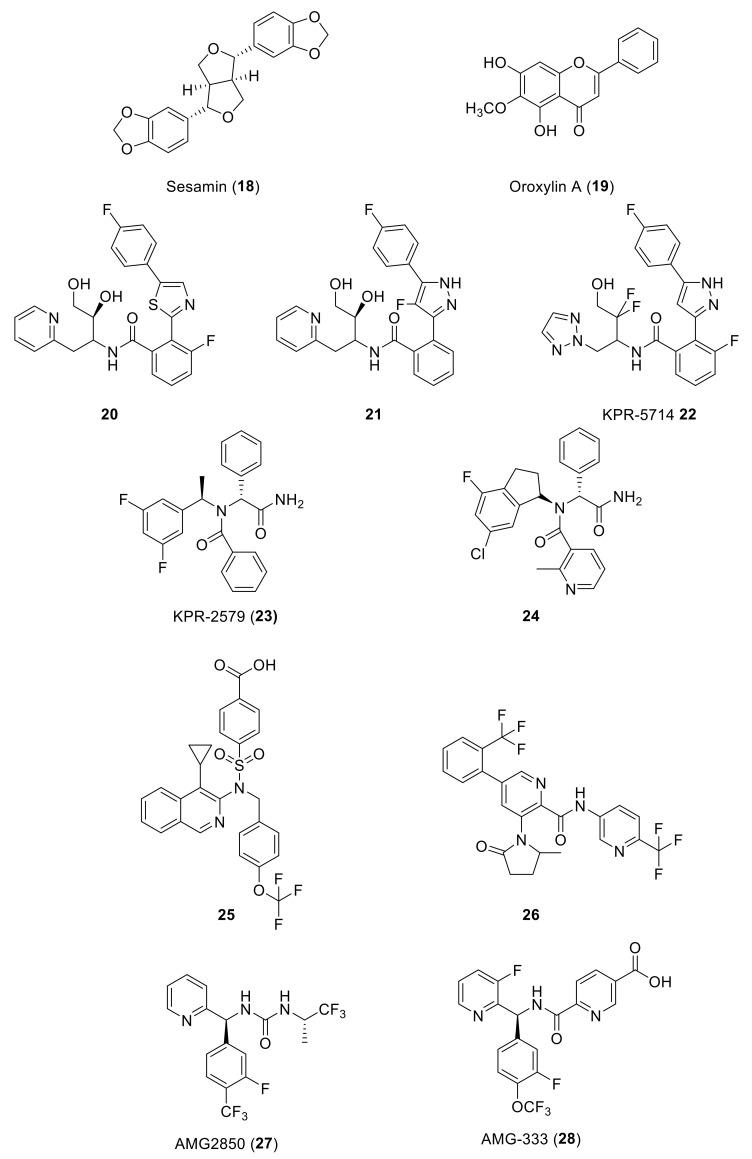
Novel TRPM8 antagonists derived from natural sources and pharmaceutical companies.

Natural products (NPs) are a continuous source of bioactive compounds, also in the TRP channels field. In recent years, two NPs have been described as TRPM8 antagonists. Derived from the traditional Chinese medicine, Yutong Sui et al. screened 158 compounds in order to identify new TRPM8 antagonists through a calcium mobilization assay [144]. Sesamin (**18**, Figure 5, Table 2), extracted from sesame, was identified as a TRPM8 antagonist with an IC_50_ value of 9.79 µM, selectivity over a panel of other six TRP channels, and no cytotoxic effect up to 90 µM. Molecular docking studies, performed on the complete TRPM8 channel, determined that sesamin forms hydrogen bonds with Arg832 and Arg998, a cation-π interaction with R832, and also hydrophobic contacts with Tyr736, Leu769, Ile797, Ile836, and Phe829. Sesamin was evaluated in prostate tumor adenocarcinoma, showing the suppression of cell proliferation in DU145 and LNCaP prostate tumor cell lines. The evaluation of different flavonoids aglycones on TRP channels afforded new TRPA1 agonists and TRPM8 antagonists [145]. Thus, flavones as chrysin, scutellarein or hispidulin, at a concentration of 10 µM, potently inhibited TRPM8 channel activation. Hispidulin has an IC_50_ value of 9.7 µM, while its analogue oroxylin A (**19**, Figure 5, Table 2) displays better inhibitory activity (IC_50_ = 1.7 µM), indicating that the hydroxyl group removal from the distal phenyl ring in **19** is an important factor for TRPM8 inhibition. 

The main source of TRPM8 antagonists are synthetic compounds, most of them identified and further optimized by pharmaceutical companies. Thus, KISSEI PHARMACEUTICAL CO described 2-(phenylthiazolyl)benzamide derivatives acting as TRPM8 antagonists [146]. Molecules within this series bear different combinations of F atoms at the phenyl rings flanking the thiazolyl moiety, and a hydroxyalkyl or dihydroxyalkyl chain important for activity (Figure 5). Disclosed compounds, such as **20**, were able to almost completely inhibit the icilin-induced wet-dog shake (WDS) in female SD rats at a 1 mg/kg (p.o.) dose. As TRPM8 channels have a role in activating mechanosensitive C-fibers in bladder afferent pathways, TRPM8 antagonists could also be a promising therapeutic target for hypersensitive bladder disorders. In this sense, compound **20** produced the elongation of a micturition interval in female rats (156%). 

Related families, containing a pyrazole central scaffold instead of the thiazole, were also protected by the same company, and evaluated in vivo in the above rat models [147,148]. Compound **21** (Figure 5) showed good efficacy in WDS and rhythmic bladder contractions in rats, moderated pharmacokinetic profile, and no cardiovascular effects. Further research in this series led to KPR-5714 (**22**, Figure 5, Table 2), which displayed potent and selective TRPM8 antagonist activity (hTRPM8 IC_50_ = 25.3 nM), and a good pharmacokinetic profile [149,150]. The inhibition rates of WDS by KPR-5714 were 29% at 0.1 mg/kg and 97% at 0.3 mg/kg. This compound, at 0.1 mg/kg, decreased soluble α-synuclein aggregates (SAAs) in C-fibers, but not in Aδ-fibers. KPR-5714 inhibited the exaggerated activity of mechanosensitive bladder C-fibers, increased the mean voided volume, and decreased voiding frequency, suggesting a possible alternative treatment for hyperactive bladder disorders. Aizawa et al. published the effects of KPR-5714 on rhythmic bladder contractions (RBCs) in normal anesthetized rats in combination with a β3-adrenoceptor agonist or an anticholinergic agent [151]. This study first confirmed that KPR-5714 did not act on the rat β3-adrenoreceptor and M3 receptors. The combination of KPR-5714 and mirabegron led to decreased frequency of RBCs, whereas tolterodine tartrate increased it, therefore, indicating different mechanisms of action for each drug. The simultaneous administration of KPR-5714 and mirabegron or tolterodine tartrate exhibited an additive decrease in voiding frequency in rats with cerebral infarction, as well as after cold exposure, where increased voided volume was also observed.

The same company patented a series of Nα-disubstituted glycinamides with low micromolar TRPM8 antagonist activity [152,153]. A first round of structure-activity relationships and further optimization of the pharmacokinetic properties (PK) resulted in KPR-2579 (**23**, Figure 5), a potent and selective TRPM8 antagonist, with in vivo efficacy in WDS and bladder contractions models in rats [154]. However, dose-escalation toxicity studies in rodents and in vitro studies showed undesirable effects. A glutathione (GSH) adduct of KPR-2579 was identified in rat bile and urine, causing unanticipated immune responses. In addition, convulsions were observed in rodents after high oral dose administration (300 mg/kg), and KPR-2579 induced expression of CYP3A4 cytochrome in the hepatic stem cell line HepaRG. Further structural modifications of KPR-2579 allowed the identification of a potential clinical candidate that avoids the formation of the KPR-2579-like glutation reactive metabolite. The substitution of the N-phenethyl group by a conformationally restricted N-indanyl group improved the inhibitory activity. This modification, along with the incorporation of a 2-Me-pyridine ring, led to compound **24** (Figure 5, Table 2) with a wide safety margin [154]. Compound **24** is a potent inhibitor of hTRPM8 channels (IC50 = 0.21 M), has excellent selectivity over other TRP channels, and does not form GSH adducts. This compound inhibited icilin-induced WDS in rats, and significantly reduced cold-induced frequent urination, at a 3 mg/kg dose.

In several patents since 2012, Mitsubishi Tanabe Pharma CO described quinoline-derived sulfonamides with potent TRPM8 antagonist activity [155,156]. More recently, the company claimed for using such compounds (i.e., **25,** Figure 5) for treating or preventing vasomotor symptoms (VMS) in females, with a decrease in the frequency after two days of treatment [157]. This compound also led to a significantly reduced core body temperature. The preparation and characterization of different crystal isomorphs for compound **25** have also been claimed, along with a pharmaceutical composition containing such a compound for the prevention or treatment of chronic pain and other TRPM8-dependent diseases [158]. This company patented, as well, a family of aromatic carboxamides with nanomolar and subnanomolar TRPM8 inhibiting activities which are useful for chronic-pain treatment, here exemplified by compound **26** [159,160]. 

As commented previously, TRPM8 is expressed in sensory neurons of the peripheral nervous system, including the nerve circuits connected to migraine pathogenesis. In this respect, Amgem Inc reported the development of a novel series of biarylmethanamide TRPM8 antagonists [161]. In the initial SAR studies, they identified derivatives as AMG2850 (**27**, Figure 5), which showed in vivo activity in a TRPM8-mediated WDS model in rats (10 mg/Kg). Conversely, this compound was ineffective in other animal models, such as inflammatory mechanical hypersensitivity and neuropathic tactile allodynia (100 mg/Kg), suggesting either that TRPM8 channels are not implicated in these pain disorders or that more potent antagonists are needed. Therefore, their efforts were directed to the search for new compounds with an increase in vitro and in vivo properties. Within a small library of amides (52 components) and ureas (203 compounds) they found that amides were well tolerated, particularly some quinolone derivatives. Further optimization around this scaffold allowed the selection of four compounds that were further characterized for rat pharmacokinetic properties and the in vivo activity in the WDS rat model, ex vivo cardiovascular safety. Two molecules progressed to rat and dog toxicology studies, with compound AMG-333 (**28**, Figure 5, Table 2) as the best tolerated after 14 days toxicologic studies. AMG-333 is a potent (IC_50_ = 13 nM) TRPM8 antagonist, selective over other TRP channels and devoid of off-target activity. Finally, AMG-333 was well tolerated in rat and dog after 28 days, and advanced to phase one human clinical trials in patients with migraine (the results are commented later). 

Another TRPM8 antagonist was developed by Aizawa et al. at RaQualia Pharma, RQ-00434739 (RQ, structure not disclosed yet). It displayed the potent TRPM8 inhibitory potency (IC_50_ = 14 nM) and selectivity over other ion channels, including TRPA1, TRPM2, TRPV1, Nav1.3, Nav1.5, Nav1.7, Cav2.2, and Cav 3.2 [162]. This compound induced the complete inhibition of icilin-induced WDS in rats and robust efficacy in rat and monkey models of cold allodynia, developed after the administration of the chemotherapeutic agent oxaliplatin. The effects of RQ were also evaluated in body temperature, normal bladder sensory function, and bladder overactivity. Deep body temperature was not affected after RQ treatment, which could be an advantage for future clinical development. In addition, RQ inhibited the hyperactivity of SAAs at C-fibers induced by menthol (**1**) and PGE3 administration. Therefore, the this novel TRPM8 antagonist, RQ, could be a promising drug to treat bladder sensory disorders, while maintaining body temperature.

**Figure 6:**
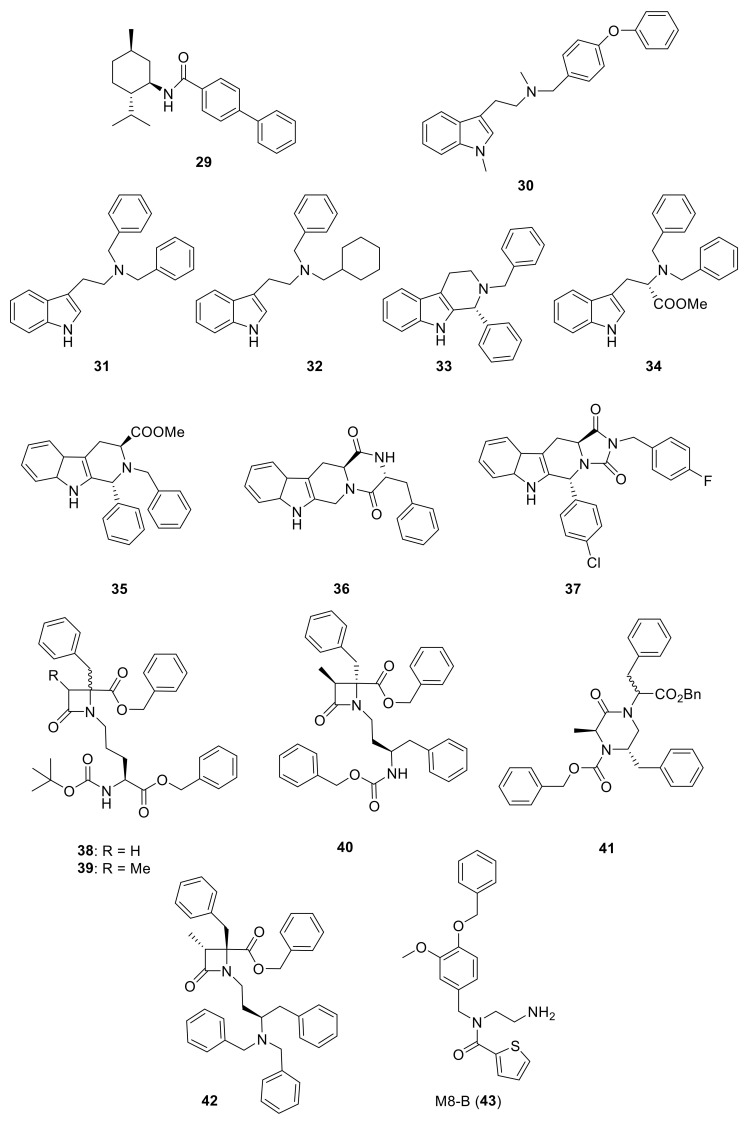
Recently published TRPM8 antagonists from academic groups.

The work of different academic groups also contributed to the identification of novel chemotypes that inhibit TRPM8 receptor activation. In a very recent paper, Journigan et al. described a (-)-menthyl derivative **29** (Figure 6, Table 2) as a potent antagonist of TRPM8 channels [163]. Calcium imaging assays were carried out to assess the (−)-menthyl **29** species-dependent inhibition of both menthol- and icilin-mediated responses, at either human or rat TRPM8, using strictly the same experimental conditions. They obtained EC_50_ values of 81 ± 17 and 107 ± 8 μM for menthol activation of human and rat TRPM8, respectively, while the EC_50_ values for icilin stimulation, were lower (526 ± 24 nM and 554 ± 12 nM at human and rat TRPM8, respectively). In both models, both agonists showed equivalent activation values. However, at *h*TRPM8 channels, compound **29** inhibited both menthol and icilin responses at higher concentrations than at the rat orthologue (*h*TRPM8: IC_50_ = 805 nM and 1.8 μM, respectively; *r*TRPM8: IC_50_ = 117 nM and 521 nM, respectively). Therefore, compound **29** could be considered a chemical tool for studying differences in TRPM8 activities between rat and human species. 

The sequence alignment of the TM domain and the TRP helix revealed that *h*TRPM8 differs from rat orthologue in 10 unique positions, and eight of them are outside the VSLD binding site. Residue Ser1007 (*h*TRPM8), corresponding to Asn1007 in *r*TRPM8, located within the TRP helix, is the only point of differentiation, which is close to the binding site in the two orthologues. The binding mode of **29** to *r*TRPM8 was explored on a homology model based on the cryo-EM structure of *fa*TRPM8 (PDB 6BPQ) [115], which shares 80% of sequence identity to *r*TRPM8. A calcium ion was placed in the VLSD, coordinated to Glu782, Gln785, Asn799 and Asp802 channel residues. Compound **29** interacted with some side-chain residues of the binding site, and the authors determined that Arg1008 residue significantly contributed to this binding. Finally, to validate initial calcium fluorescent-based assays at *h*TRPM8, they performed electrophysiology at variable concentrations of compound **29** against 500 μM menthol. In good correlation with calcium imaging experiments, measures showed that the antagonist activity of 29 at hTRPM8 was in the high nanomolar range (IC50 = 700 nM). 

In the tryptamine series, described by Gomez Monterrey’s group, compound **30**, a dimethyl derivative of the agonist **17**, and dibenzyl tryptamine **31** behaved as TRPM8 antagonists (Figure 6, Table 2) [139]. Compound **31** was rather selective for TRPM8, showing a weak activity at 50 μM on TRPV1, and unremarkable activity on TRPA1 at all tested concentrations. Derivative **30** was less selective, acting also as an antagonist of both the TRPV1 and TRPA1 channels. In Patch-Clamp electrophysiology assays, compound **31** reversibly blocked the TRPM8 channel in a concentration-dependent manner (IC_50_ = 0.37 µM). The characterization of **31** in pain perception was conducted using both an acute formalin-induced orofacial and a CCI-induced mouse pain model. Russo et al. [140] demonstrated that the systemic pre-treatment of rats with 10 mg/kg of **31** reduced formalin-induced pain. Analogously, both the systemic (1 mg/kg) and local (0.3 mg/100 μL/paw) administration of **31** reduced cold hyperalgesia induced by CCI in mice. At a 10 mg/kg dose, **31** reduced body temperature, but this effect was not significant at the effective dose of 1 mg/kg.

A SAR study on different derivatives of **31** showed that the incorporation of a methylcyclohexyl moiety in place of an *N*-benzyl group resulted in compound **32** (IC_50_ = 0.6 μM), 5-fold more effective and potent than **31** (3.2 μM), while the restriction to tetrahydro-β-carboline **33** maintained the activity in the micromolar range [164]. The replacement of tryptamine by the tryptophan scaffold led to dibenzyl tryptophan **34** (Figure 6, Table 2)**,** which was identified as a potent (IC_50_ = 0.2 nM) and selective TRPM8 antagonist (Figure 6). The chiral center plays a crucial role in the biological properties of this compound, as demonstrated by the fact that its D-enantiomer showed similar efficacy but >4000 times reduced potency (IC_50_ = 865 nM). In in vivo experiments, **34** showed significant target coverage in both the icilin-induced WDS (at 1−30 mg/kg s.c.) and the oxaliplatin-induced cold allodynia (at 0.1−1 μg s.c.) mice models. Although highly potent, compound **34** showed a short-lasting effect in both animal models, probably due to the presence of the labile ester moiety. This compound was stable to chemical hydrolysis, when dissolved in phosphate buffer at pH 7.4, but it was almost completely metabolized during a 1 h time course experiment in mice serum, generating the less active acid analog as the major metabolite [164]. 

Finally, the same group designed and evaluated a series of conformationally restricted analogues of **32** [165]. Some of these compounds, featuring chemical structures of tetrahydro carboline (Figure 6, representative example **35**), tetrahydro pyrazino[1 ′,2′:1,6]pyrido[3,4-b]indole-1,4(6H,7H)-dione (**36**), and tetrahydro-1H-imidazo-[1′,5′:1,6]pyrido[3,4-b]indole-1,3(2H)-dione (**37**, Figure 6, Table 2), showed efficient and potent TRPM8 antagonist activity according to Patch-Clamp studies (IC_50_ = 12.3, 6.57, and 4.10 nM for **35**, **36**, **37**, respectively). This exhaustive SAR study determined the nature, size, and stereochemistry more relevant for the different chemotypes activity. The most potent compounds were selective against TRPV1, TRPA1, and Nav1.7 channels and, in addition, showed an improved metabolic stability in respect to **34**. Compound **37** (IC_50_ = 4.10 nM) had a slow metabolic turnover (46.0%) in experimental conditions that involved both phase I and phase II metabolic cofactors. In animal assays, **37** (11.5 mg/kg ip) blocked the spontaneous WDS induced by icilin at both 0.5 and 2 h after injection, while 34 (10 mg/kg ip) significantly decreased the TRPM8-mediated cold hypersensitivity 0.5 h after injection, and no effect at 2 h. The imidazo-[1′,5′:1,6]pyrido[3,4-b]indole-1,3(2H)-dione derivative **37** displayed an acute antinociceptive response in the oxaliplatin-induced cold allodynia mode, but only at high concentrations (at 10–30 μg sc) and 15 min after its application. Instead, in a mice model of CCI-induced hyperalgesia, 11.5 mg/kg ip of **37** produced remarkable analgesic activity.

Modeling studies using the three-dimensional cryo-EM structure of Diver et al. [121], the TRPM8/TC-I2014 complex (PDB code 6O72) revealed that compound **34** bound within the TRPM8 S1-S4 of VSLD, adopting a particular shape in which the ligand benzyl groups establish an intramolecular π−π stacking interaction [164]. This disposition allowed the formation of other π−π interactions with several protein residues, as well as a large set of H-bond contacts that stabilized the ligand/protein complex. The indole group of **34** was involved in both π−π stacking and π−cation interactions with Tyr736 and Arg998, respectively, whereas there was an edge-to-face π−π stacking between one benzyl function and Phe729. H-bonds were detected for compound **34** with Asn732 and Gln776 channel residues. Concerning compound **37**, featuring four fused rings (tetrahydro-1H-imidazo[1′,5′:1,6]pyrido[3,4-b]indole-1,3(2H)-dione scaffold), there was a flip of the indole ring that has been attributed to the presence of a substituent at C-5, thus, allowing π−π stacking contacts interactions with Phe729 and Tyr995, as also observed for tricycles derivatives having an aryl substituent in their equivalent C-1 position. In addition, the 4-Cl-phenyl substituent at C-5 in **37** determined further π−π interaction with Tyr736, whereas an H-bond contact was established with Arg998.

Starting with a high throughput screening campaign, the groups of González-Muñiz and Ferrer-Montiel identified some β,γ-diamino ester derivatives as modulators of different TRP channels, but with a low selectivity among different channel subtypes [166]. The placement of the hydrophobic substituents in this family of the compound on a more rigid scaffold led to the discovery of β-lactam-based TRPM8 antagonists [167]. In electrophysiology assays, representative compounds **38** and **39** (Figure 6), obtained from Asp-Phe conjugates as diastereomeric mixtures, displayed IC_50_ values of 46 nM and 83 nM, respectively, and showed good selectivity against other channels. These compounds were able to block different modes of TRPM8 activation, including the temperature, voltage, and the cooling compound menthol (**1**).

A related family was described starting from Z-phenylalaninol-Phe conjugates [168]. In this case, the β-lactam ring closure through basic treatments of key intermediates afforded mixtures of the expected β-lactam (i.e., **40**, Figure 5, Table 2) and the corresponding 2-ketopiperazine (i.e., **41**, Figure 5, Table 2). The selectivity toward the formation of the 4- or 6-membered ring and the configurational purity of the obtained β-lactams were dependent on the configuration of the starting conjugates. The enantiopure compound **40** showed 0.4 and 0.8 µM IC_50_ values in Ca^2+^ fluorimetric assays and in Patch-Clamp experiments, respectively, and did not display significant activity at close TRPV1 and TRPA1 channels. Diastereoisomeric 2-ketopierazine **41** was also able to inhibit TRPM8 activation, but with a lower selectivity. In an in vivo model of oxaliplatin peripheral neuropathy, compound **40** (1μg, s.c.) significantly reduced cold-induced paw licking 15 min after administration, with maximum and sustained activity from 30 to 60 min, an antiallodynic activity that was higher or equivalent to other described TRPM8 antagonists. Compound **40**, and one of its isomers, exhibited micromolar non-selective antitumor activity in different tumor cell lines. Substitution of the benzyloxycarbonyl group in **40** by an *N,N*-dibenzyl moiety, and exploration of different configurations in these products, still provided improved antagonists, as published recently [169]. Compound **42** (Figure 6, Table 2), the most potent to date within this second generation of β-lactams, has nanomolar activity in both assays (IC_50_ = 20 and 80 nM in Ca^2+^ and Patch-Camp experiment, respectively). This study also demonstrated the importance of the configuration for potency in this family of compounds, although all prepared diastereoisomers were able to significantly inhibit the menthol-induced TRPM8 activation.

Docking simulations on a model generated from the *fa*TRPM8 structure (PDB code 6BPQ) [115], were performed with compounds **40** and **42**, among other derivatives in this series. The results of these studies suggested no occupancy of the menthol binding site (VSLD-TRPD), as calculated or experimentally established for some other TRPM8 antagonists [121,165]. β–Lactam derivatives, **40** and **42,** preferentially interacted with the channel by the pore zone. Solutions with the best binding energies for both compounds identified a binding site at the inner pore, in the middle of the transmembrane S5–S6 region of two adjacent subunits, and the S5–S6 segment, forming the pore of just one of these protomers. For example, 3*R*,4*R*,2′*R*-β-lactam **42** interconnected with channel S5 across Phe874, Trp877 and Phe881 residues, and with S6 through Thr956, Leu959, Val960, Ile962, and Tyr963 residue side-chains. Phe912, Glu914, and Val915, within the S5-S6 connecting segment, also participated in the stabilization of the complex. Interactions with Trp877 and Phe881 of the channel are T-shaped π–π stackings with *N*-benzyl substituents. The second solution in energy located compound **42** at the bottom mouth of the pore, just before the cytosolic entrance, and involved a different Van der Waals interaction with Met978, Tyr981, Thr982, and Ile985 of the four protein monomers. None of these residues have been identified as important for other antagonists and, therefore, it seems that this family of β-lactam derivatives could act differently to other model inhibitors of TRPM8-activation.

Epilepsy is a chronic condition associated with recurrent and unpredictable seizures in infants and young children; fever has been implicated as the main cause of seizures. The participation of TRPM8 channels in epileptogenesis has been reported by Zandi et al., evaluating the effect of M8-B (**43**, Figure 6, Table 2), a selective TRPM8 antagonist, in different experimental animal models of seizure [170]. Their results indicated a decrease in body temperature in rat pups at doses of 6 and 9 mg/kg and reduced febrile seizures at the dose of 9 mg/kg; thus, confirming that the TRPM8 channel has an active role in body thermoregulation. When evaluated on PTZ- and electroshock-induced seizures, antagonist M8-B resulted in a significant anticonvulsant effect in the pentylenetetrazol (PTZ)-induced convulsion model, but did not have protective effects in the other model.

**Table 2:** Representative examples of newly described families of TRPM8 antagonists.

Compd.	Structure	IC_50_ (μM)		In Vivo Inhibitory Activity	Ref.
		Ca^2+^ Assay	Patch-Clamp		
**18**	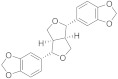	9.8	_	Antitumoral	[144]
**19**	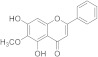	9.7	_	_	[145]
**22** SKP-5714	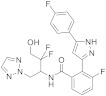	0.025	_	Cold sensibility (WDS) Bladder overactivity	[149,150]
**24**	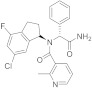	0.21	_	Cold sensibility (WDS)	[154]
**28** (AMG333)	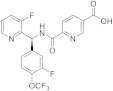	0.013	_	Cold sensibility (WDS)	[161]
**29**	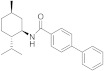	1.8	0.70	_	[163]
**31**	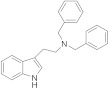	3.2	0.37	Skin hypersensitivity Neuropathic pain Cold sensibility (WDS) Reducted temperature	[139,140]
**34**	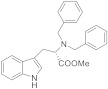	0.04	0.0002	Cold sensibility (WDS) Oxaliplatin-induced neuropathy	[164]
**37**	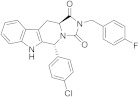	0.06	0.004	Cold sensibility (WDS) Improved oxaliplatin-induced neuropathy	[165]
**40**	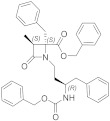	0.4	0.8	Improved oxaliplatin-induced neuropathy Antitumor activity	[168]
**41**	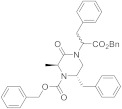	0.16	_	_	[168]
**42**	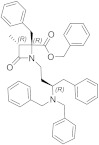	0.02	0.05	_	[169]
**43** (M8-B)	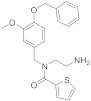	64.3	_	Decreased body temperature Epilepsy	[170]

_: Not available data.

## 6. Clinical Trials with TRPM8 Modulators

The plethora of TRPM8 agonist and antagonist families described by big pharma, small biotechs, and academic groups in the last 15 years, contrast with the few numbers of TRPM8 modulators that have reached clinical trials, excluding menthol.

The prototype of TRPM8 agonists, menthol (**1**), is the active principle in more than 220 clinical studies, including pain treatments, tobacco addition, and cosmetic applications (see a selection in Table 3). For instance, topical menthol (**1**) was evaluated in a randomized double blind, placebo controlled clinical trial (NCT02984072) for pain relief in face actinic keratosis produced after photodynamic therapy. The application of irritant *trans*-cinnamaldehyde, a TRPA1 agonist, led to cutaneous pain and hyperalgesia, while co-application with menthol suggested the beneficial effects of this TRPM8 agonist as topical antihyperalgesic (clinical study NCT02653703) [171].

Several studies deal with the application of Biofreeze topical gel (containing menthol) in different painful diseases. Recently, a phase two study started to evaluate the effect of Biofreeze on knee osteoarthritis, a limiting disease that causes intense joint pain (NCT04351594). The validity of this treatment was previously published [172], and another related clinical trial has been withdrawn (NCT01565070, Chilean authorities did not authorize the use of Biofreeze). NCT03012503 is also a clinical trial directed to know the effects of this biogel on pain neck provoked after chiropractic cervical manipulation, but no conclusions have been published to date.

Menthol (**1**) and mixtures of menthol + mannitol have also been clinically investigated (NCT01855607) for the treatment of painful chemotherapy- and diabetes-induced peripheral neuropathies, respectively, in selected breast and colorectal cancer patients (status unknown yet). A similar work was published a few years ago, in which this worsening neuropathy was relieved by menthol in a patient with colon adenocarcinoma [173].

Results from the clinical trial NCT01716767 indicated that topical menthol (**1**) reduces pain intensity in hands and arms in slaughterhouse workers suffering of carpal tunnel syndrome, offering this agonist as a non-systemic alternative to most common analgesics [174]. Recent recruitment of patients with diabetic peripheral neuropathy has started to assess if treatment with menthol (**1**), alone or in combination with mannitol, could be different in relieving pain severity in this neuropathy (NCT02728687).

The possible application of STOPAIN^®^, a topical gel containing 6% menthol (**1**), for migraine mitigation was studied in the pilot study NCT01687101 on subjects diagnosed with episodic migraine, with or without aura. A significant decrease in headache intensity after gel application was detected, suggesting that this could be an effective treatment for acute migraine attacks [175]. A recent study points to a functional cross-talk between TRPV1 and TRPM8 in trigeminal ganglion neurons as a possible mechanism for explaining how facial TRPM8 activation can suppress TRPV1 activity, relieving migraine pain [176].

Oropharyngeal dysphagia is prevalent in aged patients with neurological diseases, and leads to malnutrition and swallowing difficulties that many times result in serious aspiration pneumonia. The clinical trial NCT03050957 evaluated the possible effects of oral menthol (**1**, administered in food bolus) in restoring the swallowing dysfunction in these patients (results not been disclosed yet). The double-blind, randomized clinical trial NCT01408446 evaluated the effects of menthol (**1**) on blood pressure and other metabolic parameters in pre- and mild hypertensive patients. Main results indicated that TRPM8 activation benefits both vascular function and blood pressure, through inhibiting Ca^2+^ signaling-mediated RhoA/Rho kinase activation in blood vessels, suggesting that long-term dietary menthol treatment could have favorable effects on hypertension [177].

A menthol derivative, menthoxypropanediol, also with TRPM8 agonist activity was studied in biopsies of atopic dermatitis patients to evaluate its interest in treating pruritus associated to this pathology (NCT03610386). As described, menthoxypropanediol was effective against itch, with a fast satisfaction of patients [178]. Similarly, Cryosim-1, a triphosphine oxide TRPM8 agonist, with a heptane linear chain, was studied for the treatment of itching, with evidence that points to its potential for the immediate relief of itch [179]. A pilot study with TRPM8 agonist (Cryosim-3, **16**) in patients with dry eye neuropathic pain indicated that the topical administration of this compound relieved pain intensity, after a week of 4 administered daily doses, and an improvement of associated factors such as tearing, burning sensation, and light sensitivity [180].

D3263 compound, by Dendreon Corporation, is an oral benzimidazole-menthol derivative with TRPM8 agonist activity and good oral bioavailability, which reached phase one clinical trial in patients with solid tumors (NCT00839631). Apparently, D3263 was able to stabilize the disease in advanced prostate cancer patients, without significant cardiac toxicity [181], and in benign prostatic hyperplasia. No news on the benefits of this compound have been published since 2012, and the study seems to be discontinued.

Odorless and tasteless cooling agents, with a higher potency than menthol (**1**), are currently commercialized as flavor additives for food and beverages and as cosmetic ingredients, and companies such as Givaudan AG, BASF, Procter & Gamble, and Wilkinson have ongoing programs toward more potent and long-lasting cooling compounds, as indicated in the agonist section [182].

Naturally occurring TRPM8 antagonists include plant cannabinoids, such as cannabidivarin. This non-selective TRPM8 antagonist, with multitarget activity, including TRPV1, TRPV2 and TRPA1 antagonist activity [183], is under different clinical studies. A phase two clinical trial, NCT03849456, is currently evaluating the safety and tolerability of this cannabis-derived product in children and young adults with autism spectrum disorders. Similarly, trial NCT02369471, also in phase two, is directed to investigate the safety, tolerability, and pharmacokinetics of cannabidivarin in combination with antiepileptic drugs in patients with focal seizures.

The potent and selective TRPM8 antagonist PF-05105679, from Pfizer, reached phase one clinical trials (NCT01393652). However, trial volunteers described non-tolerated side effects, such as hot sensation in the face (especially mouth), hands and arms, which stopped further progress into advanced clinical studies [184]. Similarly, the TRPM8 antagonist AMG333, from AMGEN Inc., progressed to a phase one clinical trial (NCT01953341) in healthy subjects and patients with migraine. This compound showed a good pharmacokinetic profile, but adverse effects such as feeling hot, paresthesia, dysesthesia, and dysgeusia hindered further development, and the study was discontinued [161].

Looking for a diagnosis test, clinical trial number NCT04286542 is directed to identify mediators associated to nasal hyperreactivity after a cold, dry air influx. A selection of patients with allergic rhinitis, chronic rhinosinusitis, and healthy control subjects will be recruited. They will study the comparative presence of histamine, tryptase, and Substance P, among other biomolecules in nasal secretions, while nasal biopsies will be recruited for determining the expression of various TRP channels, including TRPM8.

In short, while clinical topical administration of TRPM8 agonists is described to relief several types of pain, including inflammatory and mechanical painful processes, as well as migraine and some peripheral neuropathies, oral treatments with both agonists and antagonists produced important secondary effects that still preclude their progression into the clinic.

**Table 3:** Selection of main clinical studies on TRPM8-targeted therapies and diagnosis probes ^a^.

Compound	Function	Route of Administration	Indication	Clinical Phase	Clinical Trial Number	Ref.
Menthol (**1**)	Agonist	Topical	Pain, after photodynamic therapy	4	NCT02984072	_
Menthol (**1**)	Agonist	Topical	Pain, after TRPA1 agonist	NA	NCT02653703	[171]
Menthol (**1**) (Biofreeze)	Agonist	Topical	Knee osteoarthritis Mechanical/Neck pain	2 Withdrawn NA	NCT04351594 NCT01565070 NCT03012503	[172] _ _
Menthol (**1**)	Agonist	Topical	Chemotherapy-induced peripheral neuropathy	2	NCT01855607	[173]
Menthol (**1**)	Agonist	Topical	Carpal tunnel syndrome	NA	NCT01716767	[174]
Menthol (**1**) + manitol	Agonist	Topical	Diabetic peripheral neuropathy	1–2	NCT02728687	_
Menthol (**1**) (STOPAIN^®^)	Agonist	Topical	Migraine	NA	NCT01687101	[175,176]
Menthol (**1**)	Agonist	Oral	Oropharyngeal dysphagia	2	NCT03050957	_
Menthol (**1**)	Agonist	Oral	Hypertension	2	NCT01408446	[177]
Menthoxypro- panediol	Agonist	Topical	Pruritus Atopic dermatitis	NA	NCT03610386	[178]
Cryosim-1	Agonist	Topical	Itch	NA	ND	[179]
Cryosim-3 (**16**)	Agonist	Topical	Dry eye disease	NA	ND	[136]
D-3263	Agonist	Oral	Cancer	1	NCT00839631	[181]
Cannabidivarin	Antagonist		Autism Epilepsy	2 2	NCT03849456 NCT02369471	[183] _
PF-05105679	Antagonist	Oral	Pain	1	NCT01393652	[184]
AMG 333 (**28**)	Antagonist	Oral	Migraine	1	NCT01953341	[161]
Diagnosys test	_	_	Cold, dry air	NA	NCT04286542	_

^a^ Source: www.clinicaltrials.gov (30 June 2021); NA: Not applicable; ND: Not disclosed yet.

## 7. Perspectives

In the last 15 years, the incessant research on TRPM8 channels has revealed their participation in numerous physiological processes and in various pathological states [95]. The future advancement in this field should necessarily occur through the progression in various directions.

Despite the number of studies focused on understanding the TRPM8 connection to cancer, forthcoming treatments with TRPM8 modulators are still in need of more coordinated investigations. For example, the identification and/or quantification of TRPM8 expression in each malignancy, and its evolution along tumor states, are essential because of the differential expression and role of TRPM8 channels among different cancer types. This also applies to other TRPM8-involved diseases and, therefore, apart from using TRPM8 as a biomarker [185], the development of diagnosis probes based on TRPM8 ligands is a subject that needs to be approached as soon as possible. Additionally, in the field of cancer research, the contribution of TRPM8 receptors to CIPN and to the repair of radiation-induced DNA damage [186] deserves further investigation.

TRPM8 modulation could also serve to alleviate pain suffering in processes that occur after inflammation, nerve injury or peripheral neuropathies [95]. However, further research should be carried out to stablish the basis of agonists and/or antagonists’ effects, as they are either not clear enough or remain a matter of controversy. In this regard, redefining pre-clinical human pain models is also required to increase clinical translation, but in the meantime, the identification of primate sensory neuron types and their protein content is contributing to the better understanding of the cellular and heritage basis of chronic pain in human related species [187]. The identification of genetic polymorphisms in TRPM8, and their connection to individual differences in pain sensitivity, could help in the way to personalized medicines [70,188]. On the other hand, gender dependence should also be considered within in vivo experimentation, especially in pain models [189].

Improved knowledge into the intercommunication of TRPM8 channels with other protein receptors, as well as with related and unrelated ion channels, is another crucial aspect to identify cellular pathways interconnected in TRPM8-linked diseases. Lately, numerous articles are being published on this respect [54,190,191,192]; thus, providing crucial information on physiological and pathological cellular pathways related to these TRP channels.

Unwanted side effects of the TRPM8 modulator found in phase one and two clinical trials should direct us either to focus on the TRPM8 stimulus-specific molecules or to “dirty” compounds synergistically acting on TRPM8 and other receptors. Until we can dissociate activity due to different stimulus, the topical or local tissue administration of TRPM8 modulators seems to be a better option than systemic distribution. Additionally, campaigns to evaluate the selectivity of TRPM8 modulators against other channels and receptors should be established in a more regular basis. In that way, selective or multitarget compounds with synergic activities could be selected for pharmacological in vivo characterization. Unfortunately, the high prices of screening companies greatly limit the recruitment by academic groups and small biotechs, but a possible solution could be the fostering of a more global collaboration among groups involved in ion channel research. It is expected that the current knowledge on TRPM8 structures [115,121,124] could encourage the rational design of new TRPM8 modulators, with improving selectivity or dual or multitarget activity. Alternative medicinal chemistry strategies could also be envisaged, such as the protein degradation PROTAC approach, which can be fueled after the identification of the tripartite motif-containing 4 (TRIM4) [87], an ubiquitin (Ub)-ligase E3 acting as an interaction partner of TRPM8 ubiquitination. Nanotechnologies could also be of help in the way of diagnosis probes and for tissue specific drug delivery. In fact, a carbohydrate polymer-based hydrogel, with a nanofiber-like structure, has already been described for a long-lasting and optimal delivery of the TRPM8 antagonist AMTB [193].

Unquestionably, many efforts have been made in recent years for advancing in different aspects related to TRPM8 channels, but we can anticipate that there are still many more things to do and a world of opportunities in front of us.

## Data Availability

Not additional data available.
